# Finished Genome of *Zymomonas mobilis* subsp. *mobilis* Strain CP4, an Applied Ethanol Producer

**DOI:** 10.1128/genomeA.00845-13

**Published:** 2014-01-09

**Authors:** Vassili N. Kouvelis, Hazuki Teshima, David Bruce, Chris Detter, Roxanne Tapia, Cliff Han, Vassileia-Olga Tampakopoulou, Lynne Goodwin, Tanja Woyke, Nikos C. Kyrpides, Milton A. Typas, Katherine M. Pappas

**Affiliations:** Department of Genetics & Biotechnology, Faculty of Biology, National and Kapodistrian University of Athens, Panepistimiopolis, Athens, Greecea; DOE Joint Genome Institute, Bioscience Division, Los Alamos National Laboratory, Los Alamos, New Mexico, USAb; DOE Joint Genome Institute, Walnut Creek, California, USAc

## Abstract

*Zymomonas mobilis* subsp. *mobilis* is one of the most rigorous ethanol-producing organisms known to date, considered by many to be the prokaryotic alternative to yeast. The two most applied *Z. mobilis* subsp. *mobilis* strains, ZM4 and CP4, derive from Recife, Brazil, and have been isolated from sugarcane fermentations. Of these, ZM4 was the first *Z. mobilis* representative strain to be sequenced and analyzed. Here, we report the finishing of the genome sequence of strain CP4, which is highly similar but not identical to that of ZM4.

## GENOME ANNOUNCEMENT

*Zymomonas mobilis* subsp. *mobilis* strain CP4, formerly known as *Z. mobilis* var. *recifensis* (1), is a most aerotolerant, quickly growing, and ethanol-yielding *Z. mobilis* strain (2, 3). CP4 and its kin strain *Z. mobilis* subsp. *mobilis* ZM4 originate from the same source at Recife, Brazil (4, 5), and are known to reach theoretical maxima of ethanol production when grown on glucose substrates (6). Both strains have undergone numerous independent genetic manipulations in order to be optimized for ligninocellulosic biomass fermentations (3, 6–8). Despite their wide applications, controversy exists as to whether these strains are different or identical; although they have distinct plasmid profiles (5), they are currently considered the same strain by bacterial repositories, including the American Type Culture Collection (ATCC 31821 [http://www.lgcstandards-atcc.org/products/all/31821]). The finishing of the genome sequence of CP4 unequivocally proves that CP4 is not identical to ZM4.

Total DNA from CP4 was prepared as described previously (9). The genome sequence was generated at the Department of Energy (DOE) Joint Genome Institute (JGI) (http://www.jgi.doe.gov/) using a combination of Sanger, Illumina (10), and 454 technologies (11). To this end, a Sanger library (average insert size of 6.6 ± 1.6 kb), a 454 Titanium standard library (395,449 reads), two paired-end 454 libraries (average insert sizes of 4.6 ± 1.1 bp and 25.1 ± 6.3 kb), and an Illumina GAII shotgun library were constructed, generating sequence reads totaling 2.1 Mb, 202.0 Mb, and 222 Mb, respectively. The 454 and Illumina data were assembled with Newbler version 2.3 and Velvet version 0.7.63, respectively (12). The Sanger reads, the 454 Newbler consensus shreds, the read pairs in the 454 paired-end library, and the Illumina Velvet consensus shreds were integrated using parallel Phrap version SPS-4.24 (High Performance Software, LLC). The software Consed (13–15) was used for finishing. The Illumina data were used to increase consensus quality using the software Polisher developed at JGI (A. Lapidus, unpublished data). Possible misassemblies were corrected using Gap Resolution (C. Han, unpublished data), dupFinisher (16), or sequencing bridging PCR fragments after subcloning. The gaps between contigs were closed by editing in Consed, by PCR, and by Bubble PCR primer walks (J.-F. Cheng, unpublished data). The final assembly is based on data providing coverage of the genome up to 106-fold. Coding gene prediction, functional gene assignment, and tRNA/rRNA identification were determined as described before (17). Genome structure comparisons relied on ACT (18), BLASTn (19), and MegaBLAST (20).

The genome of CP4 comprises a circular chromosome of 1,998,637 bp and five plasmids, pCP4_1 to pCP4_5, of 36,892 bp, 33,915 bp, 32,400 bp, 30,952 bp, and 30,440 bp, respectively (G+C contents of 46.24% for the chromosome and of 42.39%, 42.28%, 43.69%, 43.70%, and 42.68% for the five plasmids, respectively). It has 1,860 protein-coding genes, 48 tRNA genes, and 2 rRNA gene clusters.

The CP4 genome is 57,727 bp smaller than that of ZM4 (21) and shares syntenic units that locally reach 99% identity. However, four stretches within the CP4 chromosome totaling 20,452 bp (coordinates 110280 to 121208, 1243209 to 1246778, 1259989 to 1262157, and 1552432 to 1554025) and 18 genes are unique to the strain compared to ZM4; conversely, 15 regions totaling 74,674 bp and 58 genes are unique to ZM4. The CP4 plasmids harbor housekeeping and accessory genes (host-beneficial or other), as well as genes of phage origin, located on pCP4_1.

### Nucleotide sequence accession numbers.

The CP4 genome was assigned GenBank accession no. CP006818 for the chromosome and CP006891 to CP006895 for the plasmids.
